# Learning From Loss After Risk: Dissociating Reward Pursuit and Reward Valuation in a Naturalistic Foraging Task

**DOI:** 10.3389/fpsyt.2019.00359

**Published:** 2019-05-29

**Authors:** Samantha V. Abram, A. David Redish, Angus W. MacDonald

**Affiliations:** ^1^Department of Psychology, University of Minnesota Twin Cities, Minneapolis, MN, United States; ^2^Sierra Pacific Mental Illness Research Education and Clinical Centers, San Francisco VA Medical Center, and the University of California, San Francisco, San Francisco, CA, United States; ^3^Department of Neuroscience, University of Minnesota Twin Cities, Minneapolis, MN, United States; ^4^Department of Psychiatry, University of Minnesota Twin Cities, Minneapolis, MN, United States

**Keywords:** risk, regret, foraging, decision-making, externalizing

## Abstract

A fundamental feature of addiction is continued use despite high-cost losses. One possible driver of this feature is a dissociation between reward pursuit and reward valuation. To test for this dissociation, we employed a foraging paradigm with real-time delays and video rewards. Subjects made stay/skip choices on risky and non-risky offers; risky losses were operationalized as receipt of the longer delay after accepting a risky deal. We found that reward likability following risky losses predicted reward pursuit (i.e., subsequent choices), while there was no effect on reward valuation or reward pursuit in the absence of such losses. Individuals with high trait externalizing, who may be vulnerable to addiction, showed a dissociation between these phenomena: they liked videos more after risky losses but showed no decrease in choosing to stay on subsequent risky offers. This suggests that the inability to learn from mistakes is a potential component of risk for addiction.

## Introduction

Many choices, like starting a new relationship or accepting a job out of state, involve some level of risk that can be expressed as a win or loss relative to baseline (1). Such decisions can lead to negative affective experiences, particularly if an individual chooses to take a risk and then receives an unfavorable outcome (2). While some individuals learn to make choices that minimize future negative outcomes (3, 4), the inability to learn from such losses may be integral to certain externalizing psychopathologies like addiction (5, 6). In this study, we examined relations between risky losses and externalizing tendencies by modifying a newly established human foraging paradigm (the *Web-Surf Task*) (7).

An earlier version of the Web-Surf Task was based on a rodent neuroeconomic task (*Restaurant Row*) (8). These parallel tasks entailed serial stay/skip choices regarding offers of real-time delays and primary rewards (food from four feeder sites in Restaurant Row, video clips from four galleries in the Web-Surf Task). On each encounter in the Web-Surf Task, the subject was informed of a required delay before the reward would be delivered, indicated by a download bar and numeric text instruction. The subject could either accept the deal and *stay* through the delay for the reward, or *skip* the deal and try his or her luck at the next reward site (video gallery). Reward kind (genre of video) remained constant at each gallery. Subjects had a limited time to spend on the task, thus creating delay-related trade-offs between galleries. Delay was random (selected uniformly from 1 to 30 s) on each offer encounter.

In our earlier work, we observed comparable decision valuation processes across species using these analogous tasks (9). Each subject revealed different, but reliable, delay-dependent preferences (i.e., thresholds) for each restaurant/gallery, taking delays below that threshold and skipping delays above. We also observed a high correspondence between choices and consummatory responses among humans (delay thresholds related to video enjoyment ratings), and between choices and stated preferences (delay thresholds related to rankings of video galleries assessed at the end of the task) (7).

Our initial work using the original Web-Surf Task bridged cross-species models of decision-making while also demonstrating the task’s capacity to parse different valuation processes (7). A critical next step is to understand whether foraging task parameters predict meaningful individual differences, like those observed on the externalizing psychopathology spectrum (including addiction). We were motivated to use the Web-Surf Task to assess externalizing tendencies for two reasons: 1) the rodent analogue (Restaurant Row) has been used to assess the effects of different substances (i.e., cocaine and morphine) on deliberation and post-decisional commitment (6), highlighting the value of this paradigm for understanding substance use disorders. 2) Recent theories suggest that foraging models of decision-making are a promising approach for studying addiction, as these tasks measure how a subject allocates scarce resources (e.g., time) when searching for valuable goods (e.g., food, drug) (10). For instance, drug users can be conceptualized as foraging for resources in a patchy environment, e.g., smokers looking for the cheapest cigarettes (11).

To better assess for behavioral markers of addiction vulnerabilities using the Web-Surf Task, we added a risk component to the task, given accumulating evidence that risky decisions represent a vulnerability for substance use disorder (12). We then characterized risky outcomes according to prospect theory (13), which raises the possibility that subjects might reframe their enjoyment with regard to post-decisional outcomes. That is, they might reframe the outcome of an incurred risk (e.g., a win or loss) relative to the mid-point of the option, independent of whether the choice was the right option to take given the information at the time. For instance, the act of losing on a risky decision may impact video enjoyment regardless of whether their choice to stay and wait for that video was consistent with the offer’s value.

Our overarching goal for the current study was to test whether an experiential foraging task can measure addiction-relevant behaviors, following from theories that conceptualize risky substance use within foraging models (14). More specifically, we aimed to determine 1) whether subjects showed differential responses to risky losses with respect to their enjoyment of reward and acceptance of subsequent risky deals, and 2) whether individual differences in response to risky losses predicted variation in trait-level externalizing, a risk factor for substance use disorders (15–17). We expected bad outcomes to reduce one’s likelihood of accepting subsequent risky offers and for this pattern to be reversed among high-externalizing subjects (suggesting continued risk-taking despite negative outcomes).

## Methods

### Subjects

One hundred five undergraduate students (81% female, average age 20.2 years) from the University of Minnesota completed the current study and received compensation in the form of extra credit towards psychology courses. We targeted a sample size of around 100 subjects for our individual differences analyses (i.e., relations with externalizing scores), given an *a priori* power analysis indicating the need for 84 subjects to have 80% power for detecting a moderate effect size of *r* = 0.3 when employing a 0.05 criteria for statistical significance (based on a meta-analysis indicating small to moderate effect sizes for risk-taking and externalizing trait correlations) (18). The racial/ethnic breakdown of the sample was as follows: 63% Caucasian, 26% Asian, 4% Black/African American, 3% Hispanic, 1% American Indian/Alaskan Native, 1% Native Hawaiian/Pacific Islander, 2% other. The University of Minnesota Institutional Review Board approved the study procedures, and all subjects provided written informed consent.

### Experimental Design

**Figure 1:**
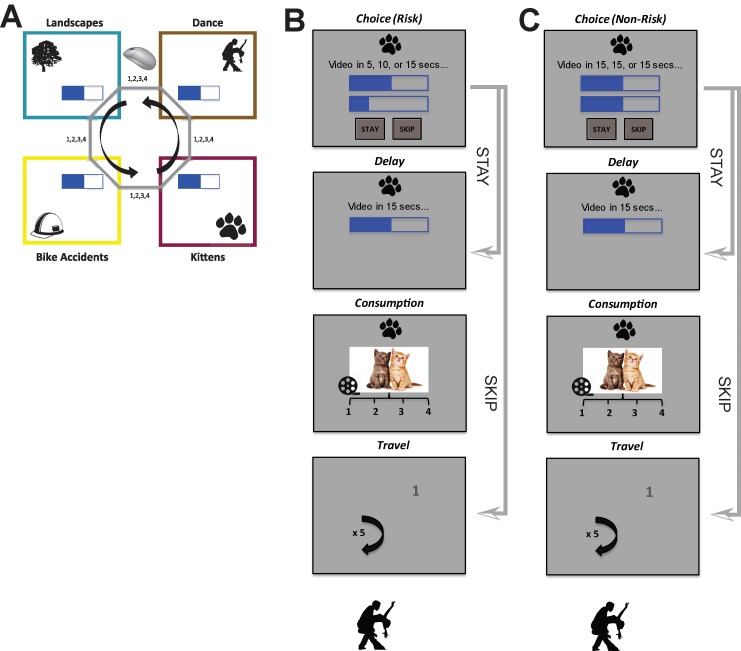
Overview of task layout. **(A)** Schematic representation of the Web-Surf Task. Subjects cycled between four video galleries (kittens, dance, landscapes, bike accidents) in a constant order. **(B, C)** Flow diagram illustrates sequencing between risky **(B)** and non-risky **(C)** trials. For a risky trial, the true delay was only revealed if the subject stayed. If they instead skipped, they advanced directly to the travel task before encountering the next offer. The travel task entailed clicking the numbers 1–4 as they appeared around the screen (traveling required five random number selections).

In the risk variant of the Web-Surf Task (**Figure 1A**), subjects had 40 min to travel between galleries that provided video rewards from the four galleries described in Abram et al. (7): kittens, dance, landscapes, and bike accidents. As in the original Web-Surf and Restaurant Row tasks, subjects had a fixed amount of time to forage; this means that subjects should have made economically maximizing decisions and stayed when the subjective value of an offer exceeded its cost.

Subjects encountered serial offers that presented a set of possible delays (**Figure 1B, C**): on entry into a gallery, the subject was shown a gallery icon, a textual representation of the offer, a pair of web-page like delay bars showing the maximum and minimum delays that could be received on that trial (possible delays ranged from 3 to 30 s), and the option to wait through the delay for a video from that gallery or move on. If the subject chose to wait, the actual delay was revealed, the delay counted down, and a 4 s video was shown; the subject then rated the video from 1 to 4 as an indicator of how much he or she liked it (4 = highest). Enjoyment ratings were made with key presses, and the task did not proceed until subjects input a rating (thus, there were no missing ratings). Importantly, in this version of the task, punishment was inescapable: subjects were locked in after making a stay choice (after which the delay began to count down). After each trial (regardless of the choice to stay or skip), the subject had to perform a short “travel” task, which entailed clicking the numbers 1 to 4 (presented in a darker shade of gray) as they randomly appeared around the screen (shown in a lighter gray). This travel task produced a cost to leaving an offer before getting to the next offer and was analogous to the travel time required as rats move between feeders during Restaurant Row.

Risky and non-risky trials were intermixed. Risk level was reflected by the variance of an offer and was either 0 (non-risky) or greater than 0 (risky, see **Figure 1**). *Risky trials* consisted of an offer with a range of delays (e.g., 5, 10, or 15 s), and each offer varied according to the set of possible delays and spread between the shortest and longest delay. (We did not allow for non-integer mid values in the risky trials, e.g., “Video in 5, 5.5, or 6 secs…” could not occur.) Critically, for risky trials, the true delay was only revealed if the subject elected to stay. Subjects were not informed of the probabilities associated with receipt of the different delays on risky trials. In comparison, *non-risky trials* presented offers with three identical delays, e.g., “Video in 7, 7, or 7 secs…”

We further classified risky trials as good or bad based on their outcome: receipt of the low delay on a risky trial was a “good” outcome, while receipt of the high delay was a “bad” outcome. (Following the framing effects from prospect theory, our definitions derive from an offer’s outcome *type* but not *value*, meaning that a bad outcome could have a delay below one’s threshold.) We were particularly interested in situations where the subject accepted a risky offer and received the bad outcome, i.e., the subject took a risk and “lost.” We contrasted these trials with a control condition, in which the subject accepted a non-risky offer of equivalent value, and with situations characterized by relief, where the subject received the good outcome on a risk trial, i.e., the subject took a risk and “won”. Importantly, the decision to *stay* or *skip* the offer on a non-risky trial, in which the true offer delay is known, can be assumed to be economically valid (i.e., correctly judged, not a mistake).

All subjects first underwent a training phase that entailed eight practice trials (two cycles through all four galleries, presented in the same order as the main task). After completion of the training phase, the subject had the opportunity to ask questions of the examiner before advancing to the main test phase.

### Trait-Level Externalizing Measure

Subjects completed the 100-item version of the Externalizing Spectrum Inventory (ESI; 19), which has been employed in several studies of undergraduate students (20–23). This inventory captures a range of traits and behaviors associated with the externalizing spectrum of psychopathology, including general disinhibition processes (e.g., theft, irresponsibility), substance use/abuse, and callous aggression.^1^ Total ESI scores were acquired by summing across all items in the inventory (20) and then applying a log-transformation to improve normality.

To assess whether behavior on the risk variant of the Web-Surf Task was related specifically to substance abuse tendencies versus externalizing behavior more broadly, we computed the three ESI subfactors: *general disinhibition* (which captures impulsivity and irresponsibility), *substance abuse* (which captures recreational and problematic substance use), and *callous aggression* (which captures physical/relational aggression and lack of empathy) (21, 24). Lastly, we computed three subscales from the substance abuse subfactor that measure problems associated with substance use: *alcohol problems*, *marijuana problems*, and *drug problems*; here, our aim was to further explore whether task behaviors predicted substance-related consequences or harms. Examples of questions in these subscales are: “My drinking led to problems at home,” “I’ve broken the law to get money for drugs,” and “At times, marijuana has been more important to me than work, friends, or school.” Because many subjects were non-responders on the problem scales, we encountered a zero-inflation problem. We thus isolated subjects who endorsed at least one item on the subscale, as individuals already experiencing negative consequences (evidence of behavioral disinhibition) are at greater risk for developing an alcohol or substance use disorder (25); 22 subjects (21%) were retained for the alcohol problem subscale analyses, versus 18 subjects (17%) for the marijuana problem subscale analyses, and 19 subjects (18%) for the drug problem subscale analyses.

### Analyses

#### Specialized Procedures

**Heaviside step function:** a piecewise function denoted H(*x*), where H(*x*) = 0 for x < 0, H(*x*) = ½ when x = 0, and H(*x*) = 1 for x > 0. This function captures the point at which a signal switches from 0 to 1. We used this function to identify the point at which subjects reliably began to skip offers (which we refer to as *delay thresholds*; see below for details). We used a Heaviside step function as an alternative to the logistic fit function described in Abram et al. (7), as the Heaviside approach is better equipped to handle extreme cases (i.e., when a subject stayed or skipped all offers in a gallery). In such instances, the Heaviside step function produces a reasonable value (e.g., the minimal or maximal delay offered), whereas the logistic function can produce values approaching infinity.

**Subject-specific delay thresholds** were computed separately for each trial using a leave-one-out approach; this yielded four thresholds, one per gallery. Thresholds were indicative of revealed preferences, reflecting the delay time at which a subject reliably began to skip offers for a particular gallery. To obtain the threshold for trial *i*, we fit a Heaviside step function to all trials in gallery *x* excluding trial *i*. This produced a vector of thresholds with length equal to the number of trials in gallery *x*. Importantly, thresholds were computed using the mid value of each offer for risky trials only. Non-risky trials were then assigned a threshold equal to the mean of the threshold vector for the respective gallery.

**Expected value for non-risky trials (with a given delay):** defined as the difference between the gallery-specific threshold and the offered delay. **Expected value for risky trials:** calculated as the average expected value of the three delays, assuming an equal likelihood for each delay (low, mid, high; see **Figure 1C**). For simplicity, we assumed a linear difference. Values ranged from −27 to 27, with a value of 0 meaning that the delay offer was equivalent to the revealed threshold.

**Mixed-effects models:** We used linear mixed-effects models to assess for group-level effects; all reported models include original p-values as well as false discovery rate (FDR)—adjusted p-values using Benjamini and Hochberg’s FDR control algorithm (26). We fit models using the MCMCglmm package in R (27), which uses Markov chain Monte Carlo techniques (see below), and lmer and lsmeans, which provided nearly identical estimates, for plotting (28, 29). The tilde (∼) in all regression models can be read as “is modeled as a function of” (30).

**Markov chain Monte Carlo (MCMC) techniques:** an approach that uses random sampling to approximate the posterior distribution of a variable of interest within a probabilistic space.

#### Validity Analyses

We evaluated the *external and face validity* of the risk variant of the Web-Surf Task using methods described in Abram et al. (7). For each subject, for each gallery, we averaged the vector of delay thresholds produced using the leave-one-out method described above; this yielded four thresholds per subject. We measured external validity by correlating delay thresholds with stated preferences (i.e., average gallery ratings and post-test gallery rankings) and obtained two validity correlations per subject.

#### Group-Level Choice, Rating, and Reaction Time Models

Our *primary choice/rating models* evaluated the impact of framing (i.e., good/bad outcome) on risk seeking (i.e., subsequent choices) and reward valuation (i.e., immediate video enjoyment ratings).

The *primary choice model* evaluated whether the type of outcome on the previous trial influenced subsequent risk seeking or aversion. This model included choice at the current trial as the dependent variable, actual value received and outcome type at the previous trial as fixed-effect independent variables, and subject as a random effect: [*Choice_t_* ∼ *actual value_t-1_* + *outcome type_t-1_* + *(1|subject)*]. This model included risky trials where the subject stayed and also received a risky offer at the next trial.

The *primary rating model* assessed the impact of framing effects on immediate reward valuation and included mean-centered rating as the dependent variable (i.e., centered to the average of the respective gallery), actual value and outcome type at the previous trial as fixed-effect independent variables, and subject as a random effect: [*Rating_t_* ∼ *actual value_t_* + *outcome type_t_* + *(1|subject)*]. This model included risky trials for which the subject stayed.

Lastly, we computed a *secondary group-level model* to examine direct relations between risk seeking/aversion and reward valuation, while considering the effects of framing and risk. In particular, we were interested in whether affective responses interacted with actual value or offer type when predicting subsequent decisions (building off the prior choice model detailed above). This model included choice at the current trial as the dependent variable; actual value, mean-centered rating, and outcome type of the previous trial, and two interaction terms as fixed-effect independent variables; and subject as a random effect: [*Choice_t_* ∼ *actual value_t-1_* + *rating_t-1_* + *outcome type_t-1_* + *actual value_t-1_:rating_t-1_* + *actual value_t-1_:outcome type_t-1_* + *(1|subject)*]. In this model, outcome type coded good outcomes, bad outcomes, and non-risky offers; this metric then reflected the framing and risk manipulations.

To assess whether bad outcomes influenced the speed at which subjects made subsequent decisions, we tested a *supplemental reaction time model* that included logged reaction times as the dependent variable, actual value received and outcome type at the previous trial as fixed-effect independent variables, and subject as a random effect: [*logRT_t_* ∼ *actual value_t-1_* + *outcome type_t-1_* + *(1|subject)*].

#### Global Risk-Aversion Trend and Control Models

We also constructed a set of models to investigate global trends in risk seeking/aversion and reward valuation, i.e., address the possibility that any trial-by-trial effects were better explained by cross-session effects. The *global risk-aversion model* included choice as the dependent variable; number of videos viewed (i.e., consumed up to trial *t*), expected value, a risky/non-risky categorical indicator, and a video consumption × risky/non-risky interaction term as the fixed-effect independent variables; and subject as a random effect: [*Choice_t_* ∼ *number videos consumed_t_* + *expected value_t_* + *risky/non-risky_t_* + *number consumed videos_t_:risky/non-risky_t_* + *(1|subject)*]. All trials were included in the choice model.

The global risk-aversion rating model was structurally equivalent to the first but included mean-centered ratings as the dependent variable: [*Rating_t_* ∼ *number videos consumed_t_* + *expected value_t_* + *risky/non-risky_t_* + *number consumed videos_t_:risky/non-risky_t_* + *(1|subject)*]. Only stay trials were included in the rating model, as subjects only rated videos during stay trials.

Based on the results of the global trend models above, we constructed a *control model* to assess whether any trial-by-trial effects were better explained by other risk-aversion patterns. Within this model, we controlled for global risk-aversion trends (number of videos consumed), as well as categorical (high, low, mid) and continuous (0–30 s) risk dimensions. Our intention was to determine if cross-session declines in accepting risky deals and/or the general tendency to prefer offers with lower risk, i.e., a more narrow offer window, could better account for the sequential choice effects seen. The control model was structured as follows: [*Choice_t_* ∼ *actual value_t-1_* + *outcome type_t-1_* + *number videos consumed_t_* + *risk_t_* + *(1|subject)*].

#### Subject-Specific Choice and Rating Models

To examine individual differences, we fit subject-specific models based on the main choice and rating group-level models. For the *subject-specific choice models*, we included choice at the current trial as the dependent variable and actual value and outcome type of the prior trial as the independent variables: [*Choice_t_* ∼ *actual value_t-1_* + *outcome type_t-1_*]. We extracted the unstandardized outcome-type coefficient that reflected the subject’s likelihood to stay following receipt of the good versus bad outcome, with higher values indicating an increased tendency to stay after receiving the bad outcome.

For the *subject-specific rating models*, we included mean-centered ratings as the dependent variable and actual value and outcome type of the prior trial as independent variables: [*Rating_t_* ∼ *actual value_t-1_* + *outcome type_t-1_*]. We again extracted the unstandardized outcome-type coefficient for good versus bad outcomes, with higher coefficients reflecting better ratings for the bad versus good outcome.

We correlated the subject-specific coefficients with trait-level externalizing, using robust partial correlation methods to reduce the influence of outliers and control for age, sex, and ethnicity. We included the age and sex demographic covariates based on prior research linking these variables with self-report and behavioral impulsivity measures (31), and more broadly with externalizing tendencies (32–34). We also included race/ethnicity, as substance use trajectories through young adulthood may differ by this factor (35). Our *primary partial correlations* related the two subject-specific coefficients with total ESI scores (distributions shown in **Figure 7**), and *follow-up partial correlations* assessed for associations with the substance abuse subfactor and subscales.

#### Delay-Discounting Comparison Models

Given the extensive literature using traditional binary choice tasks to evaluate externalizing and impulsivity (36–38), we tested whether metrics from a computerized monetary delay- and probability-discounting paradigm better explained individual differences in externalizing.^2^ This entailed subjects making a series of binary choices between hypothetical monetary rewards of different reward magnitudes associated with different temporal delays (e.g., “Would you prefer $5 now or $10 in two weeks?”) or probabilities (e.g., “Would you prefer $5 for sure or $10 with a 75% chance?”). Offers ranged from 50 cents to $10. The task lasted approximately 10 min.

A discounting rate (or k-value) was computed for the delay and probability trials separately using a hyperbolic function (39), yielding two k-values per subject. Higher k-values reflect more rapid discounting of delayed rewards and have been linked with impulsivity and addiction (40). For each subject, we checked for nonsystematic data using criteria outlined by Johnson and Bickel (41), and an R^2^ value was calculated to determine how well the data points fit the hyperbolic function.^3^ The median R^2^ was 0.86 and 0.91 for the delay- and probability-discounting rates (i.e., logged k-values), respectively. Logged parameter distributions of k from the delay-discounting experiment showed median = −5.26 days^-1^, SD = 2.05, and from the probability experiment showed median = 0.28% chance^-1^, SD = 0.84. These results are comparable to those reported in a large sample of healthy adults (31) and suggest that, for our sample, a $10 reward would be generally worth $9.52 after a 10-day delay or $8.75 when equated with a 90% chance.

## Results

### Subjects Were Willing to Wait for Videos and Showed Individual Preferences

**Figure 2:**
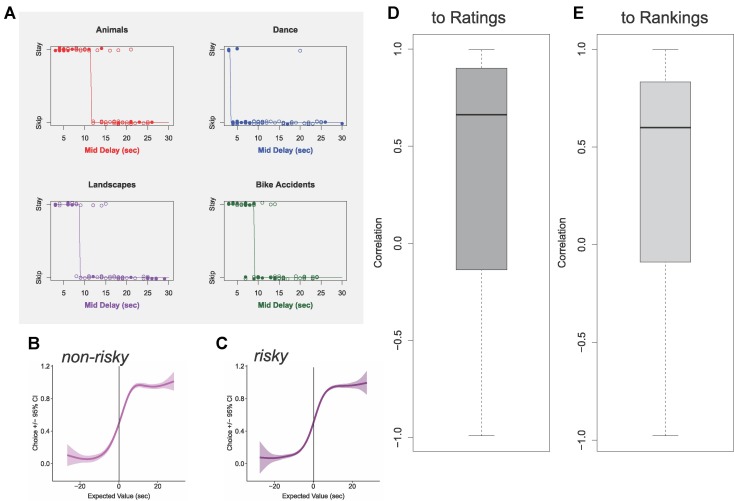
Thresholds reveal valuations. **(A)** Example thresholds identified for a typical subject. Open circles show risky trials; closed circles show non-risky trials. Threshold marked with solid line. **(B)** Average psychophysics curve for non-risky decisions, aligned to threshold for each video gallery for each subject. **(C)** Average psychophysics curve for risky decisions, aligned to threshold for each video gallery for each subject. Panels **(B)** and **(C)** are aligned to the same threshold, calculated for each gallery for each subject. **(D)** Distribution of observed correlations between revealed thresholds and video ratings. **(E)** Distribution of observed correlations between revealed thresholds and post-task stated rankings.

Subjects performed similarly on this task to what was seen in the original Web-Surf Task (7). As shown in **Figure 2D and E**, subjects showed reliable thresholds that were generally correlated with ratings (median *r* = 0.66) and with rankings (median *r* = 0.60). The decision curves of both risky and non-risky decisions depict the expected sigmoid shape, where subjects typically skipped low-valued offers (i.e., expected value < 0) and stayed for high-valued offers (i.e., expected value > 0).

### Loss After Risk Influences Choice and Reward Valuation

**Figure 3:**
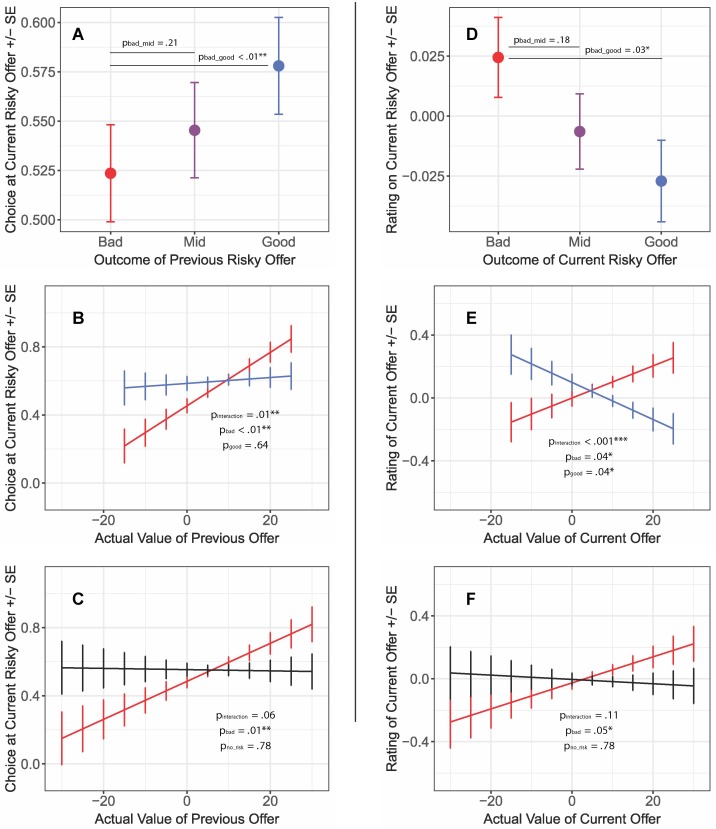
Group- and individual-level effects of risky losses on deliberation and reward likability. **(A)** Proportion of stay choices on current risky offers following receipt of the good, bad, or mid outcome on the previous risk trial. Red represents a bad outcome after accepting a risky offer; blue indicates a relief-inducing situation (good outcome after accepting a risky offer), with higher values indicating an increased likelihood of staying. Subjects were more risk-averse after risky losses. **(B, C)** Interactions between previous outcome type and actual value when predicting choices on subsequent risky offers. Black represents the control condition (equivalently valued non-risk offers). Subjects became risk-averse following risky losses of low value, versus risk seeking after risky losses of high value (whereas no associations between value and choice were detected for the relief and control conditions). **(D)** Mean-centered likability ratings following the receipt of the good, bad, and mid outcomes on the current risk trial. Subjects rated videos that followed bad outcomes more highly than those that followed good outcomes. **(E, F)** Interactions between previous outcome type and actual value when predicting immediate likability ratings (mean-centered). After a risky loss, subjects tended to rate videos that followed a low-value offer worse than those that followed a high-value offer; the inverse pattern was found for videos linked to good outcomes. A similar pattern emerged when comparing bad outcomes and control trials. Error bars indicate within-subject standard errors. *p < 0.05; **p < 0.01; ***p < 0.001.

**Figure 4:**
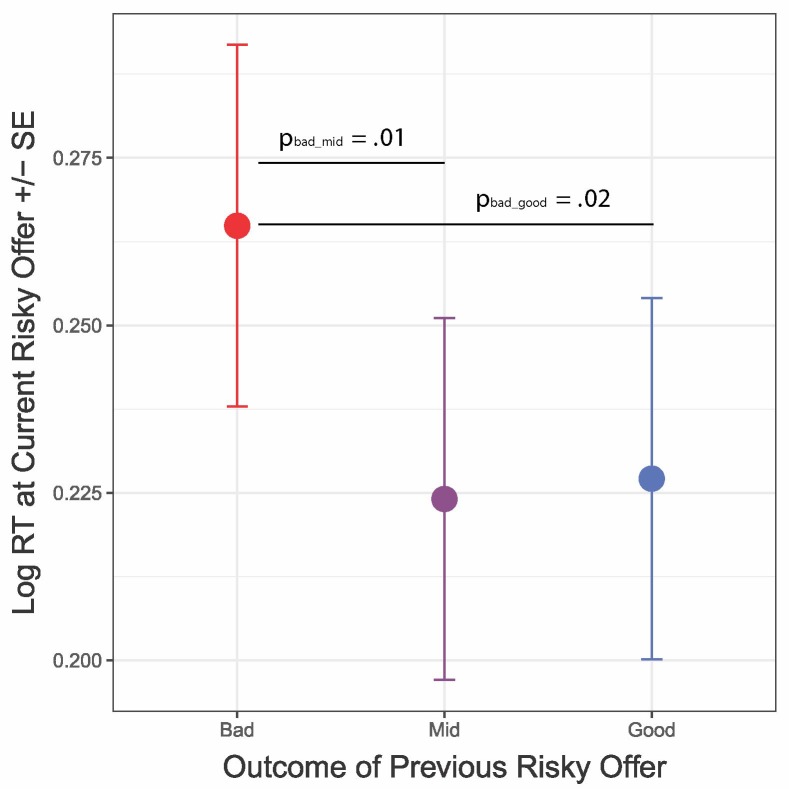
Loss after risk influences reaction times. Receipt of the bad (long-delay) outcome (red) resulted in slower reaction times (log RT) on subsequent trials, as compared to other risky conditions. Error bars represent within-subject standard errors.

To address questions of how subjects responded to loss after risk, we examined how risky outcomes impacted decision behaviors and video ratings. Here, a given delay was framed as good, bad, or in-between (mid) depending on its placement within an offer on a risky trial. Note that the true delay was known at the outset of the non-risky trials but was only revealed after the decision to stay on risky trials. Our *primary choice model* shows that, when controlling for actual value, subjects were less likely to accept a successive risky offer if they previously received a bad outcome than if they had previously received a good outcome (*p*-adj = 0.01; **Figure 3A**; **Table 1a**). Subjects were also slower to make decisions following receipt of the bad outcome (**Figure 4**; **Table 2**), suggestive of post-error slowing in response to risky losses.

**Table 1:** Choice/rating by framing models.

Predictor variable	B	CI	*P*-value	*P*-adj
(a) Choice by framing (main)				
Actual value	−.006	[−.008, −.004]	**.001**	**.002**
Outcome type (bad vs. good)	−.055	[−.098, −.020]	**.008**	**.01**
Outcome type (mid vs. good)	−.033	[−066, .005]	.10	.10
(b) Choice bad vs. good framing (follow-up)				
Actual value	.013	[.004, .023]	**.004**	**.008**
Outcome type (bad vs. good)	.134	[.010, .236]	**.02**	**.02**
Actual value × outcome type	−.014	[−026, −.002]	**.01**	**.02**
(c) Choice by bad vs. non-risk framing (follow-up)				
Actual value	.011	[.002, .019]	**.02**	**.03**
Outcome type (bad vs. non-risk)	.056	[−.048, .144]	.24	.24
Actual value × outcome type	−.010	[−.020, .000]	.06	.08
(d) Rating by framing (main)				
Actual value	.002	[.000, .004]	**.04**	**.05**
Outcome type (bad vs. good)	.051	[.005, .100]	**.03**	**.05**
Outcome type (mid vs. good)	.020	[−.022, .065]	.38	.38
(e) Rating by bad vs. good framing (follow-up)				
Actual value	.010	[.000, .020]	**.04**	.08
Outcome type (bad vs. good)	.100	[-.046, .244]	.17	.23
Actual value × outcome type	−.022	[−.038, −.009]	**<.001**	**.004**
(f) Rating by bad vs. non-risk framing (follow-up)				
Actual value	.008	[.000, .016]	**.04**	.37
Outcome type (bad vs. non-risk)	.021	[−.087, .128]	.68	1.00
Actual value × outcome type	−.010	[−.021, .004]	.11	.48

B, unstandardized coefficient; CI, confidence interval; P-adj, FDR-adjusted p-values.

Bolded text indicates p-values that are below 0.05.

**Table 2:** Logged choice reaction time by framing model.

Predictor variable	B	CI	*P*-value	*P*-adj
Actual value	.001	[−.001, .003]	.33	.44
Outcome type (bad vs. good)	.041	[.008, .071]	**.02**	**.04**
Outcome type (mid vs. good)	.001	[−.028, .032]	.95	.95

B, unstandardized coefficient; CI, confidence interval; P-adj, FDR-adjusted p-values.

Bolded text indicates p-values that are below 0.05.

*Follow-up models* clarified these sequential choice effects using subsets of trials matched by the actual value of the previous trial. The first subset included trials for which subjects stayed and received the good or bad outcome on a risky trial and encountered risk on the following trial. The second subset included trials for which subjects stayed and received the bad outcome or stayed on a non-risky trial and encountered risk on the subsequent trial. Trials were matched on a subject-by-subject basis and then combined for the group analysis. Because each subject’s contributing trials only included a portion of the possible values, we included actual value as a nested variable in the following model: [*Choice_t_* ∼ *actual value_t-1_* + *outcome type_t-1_* + *actual value_t-1_:outcome type_t-1_* + *(actual value_t-1_|subject)*]. We included the interaction term to test whether framing effects differentially shaped value-by-choice sequencing effects.

For the subset that matched bad- with good-outcome trials, we observed a significant outcome-by-value interaction (*p*-adj = 0.02; **Table 1b**); further analyses revealed that the negative framing of the previous outcome impacted relations between value of the previous trial and choice on the current trial (β = 0.014, CI = [0.004, 0.023], *p* = 0.004; **Figure 3B**). That is, subjects became risk-averse after receiving a bad offer of lower value and risk seeking after a bad offer of higher value. In contrast, we did not detect an association between the previous trial’s value and successive choice after receipt of a good outcome (β = 0.002, CI = [−0.007, 0.011], *p* = 0.64). We identified a similar (but trend-level) effect for the subset that matched bad outcome with equivalent non-risk offers (outcome-by-value interaction, *p*-adj = 0.08; **Table 1c**); follow-up analyses indicated a positive association between value and choice following receipt of a bad outcome (β = 0.012, CI = [0.004, 0.020], *p* = 0.012; **Figure 3C**), versus no association for non-risky decisions (β = 0.001, CI = [−0.007, 0.009], p = 0.78). Together, these results suggest that receipt of negatively framed outcomes (or losses), in particular, changed subsequent reward pursuit and decision-making.

But to what extent do losses after risk impact the liking of a reward? Experiments have suggested that subjects take expended costs into account when making valuations (42, 43). To address this question, we tested the impact of framing on ratings. We observed an opposite pattern in the *primary rating model* as compared to the *primary choice model*: where subjects rated videos that followed a *bad* outcome more highly than those that followed a good outcome (*p*-adj = 0.05; **Figure 3D–F**; **Table 1d**). We clarified these rating effects using *follow-up* matched-trial *models* that compared ratings that followed good versus bad outcomes and ratings that followed bad outcomes versus non-risky offers. We then fit the following model: [*Rating_t_* ∼ *actual value_t-1_* + *outcome type_t-1_* + *actual value_t-1_:outcome type_t-1_* + *(actual value_t-1_|subject)*].

These follow-up analyses revealed an interaction between actual value and rating for bad versus good outcomes (*p*-adj = 0.004; **Figure 3E**; **Table 1e**), with bad outcomes yielding a positive association between value and rating (β = 0.010, CI = [0.000, 0.020], *p* = 0.04) and good outcomes a negative association (β = −0.012, CI = [−0.022, −0.001], *p* = 0.04). Although not significant (interaction term in **Table 1f**), we saw a similar pattern for the interaction between risky and non-risky trials, with risky trials having a more substantial impact on the relationship between bad outcomes and ratings than non-risky trials (bad outcomes: β = 0.008, CI = [0.000, 0.017], *p* = 0.05; non-risky: β = −0.001, CI = [−0.010, 0.007], *p* = 0.78; **Figure 3F**).

#### Global Trends Impacted Choices But Not Ratings

**Figure 5:**
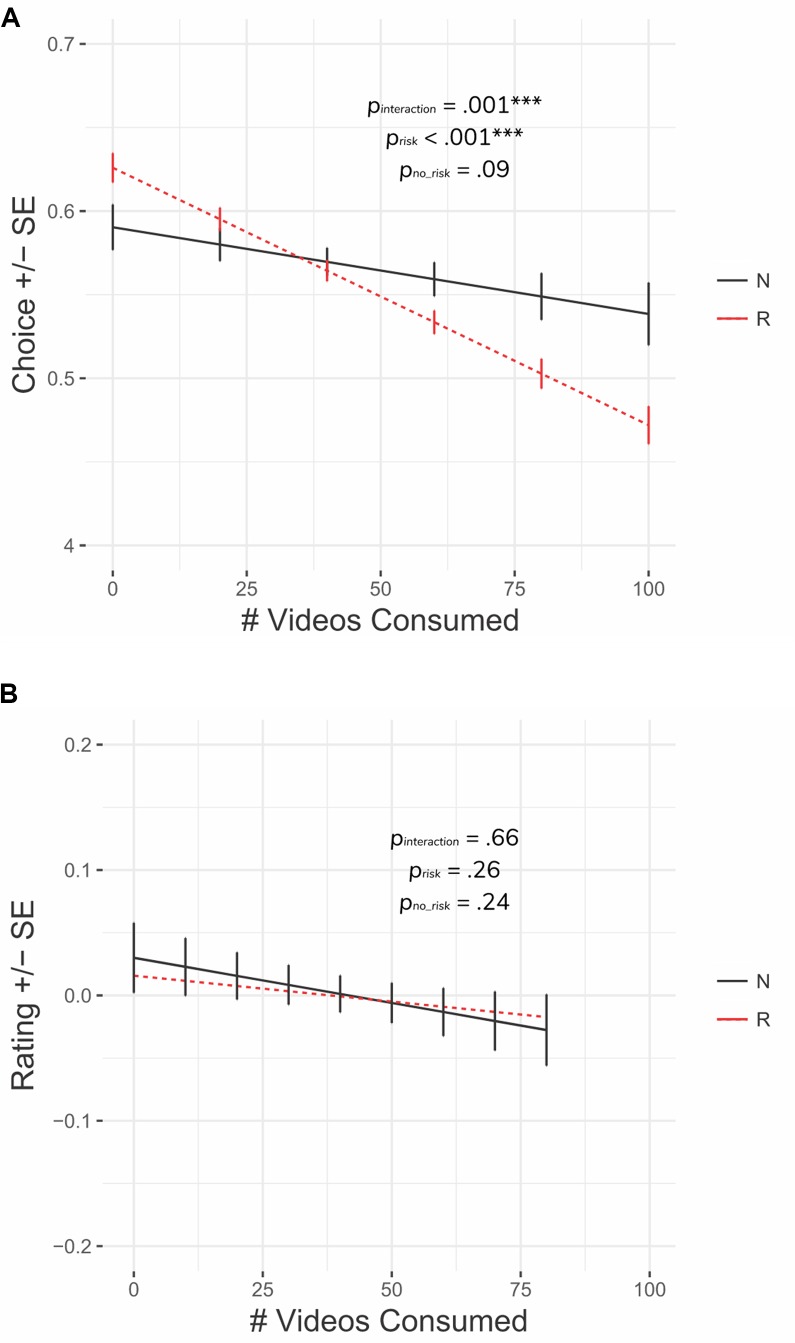
Global risk trends. **(A)** Subjects became more risk-averse as the task progressed. **(B)** Subjects’ likability ratings decreased over time but did not differ between risky and non-risky offers. Error bars represent within-subject standard errors. ***p < 0.001.

We found that subjects were less likely to accept a risky offer versus a non-risky offer as they consumed more videos (significant number of consumed videos × risk interaction, *p*-adj = 0.004; **Figure 5A**; **Table 3a**); that is, subjects became more risk-averse across the session. This interaction remained significant if the consumption variable was replaced with the number of good outcomes or bad outcomes, suggesting that this effect was not solely driven by accumulated negative experiences (but rather, risky rewards became progressively less effective in eliciting reward seeking with ongoing exposure). In comparison, we did not observe a consumption history x risk interaction (*p*-adj = 0.67) for the rating model (**Figure 5B**; **Table 3b**), suggesting that ratings were less impacted by these factors.

**Table 3:** Choice/rating by consumption models.

Predictor variable	B	CI	*P*-value	*P*-adj
(a) Choice				
# videos consumed	−.001	[−.001, .000]	**.07**	.17
Expected value	.032	[.031, .033]	**.001**	**.004**
Risk/non-risk	.036	[.007, .061]	**.006**	**.02**
# videos consumed x risk/non-risk	−001	[−.002, .000]	**.001**	**.004**
(b) Rating				
# videos consumed	−.001	[−.002, .001]	.25	.62
Expected value	.002	[.000, .004]	**.02**	.11
Risk/non-risk	−014	[−.086, .048]	.67	.67
# videos consumed x risk/non-risk	.000	[–.001, .002]	.66	.67

B, unstandardized coefficient; CI, confidence interval; P-adj, FDR-adjusted p-values.

Bolded text indicates p-values that are below 0.05.

#### Sequential Choice Effects Remained When Accounting for Global Trends

Based on the evidence that subjects grew risk-averse across the session, we built a *control model* to test whether the global risk-aversion trends (noted above) confounded trial-by-trial framing effects. The control model indicated that trial-by-trial choice effects were not better explained by consumption history (i.e., number of videos consumed) or risk level (i.e., spread of delays on a risky offer; **Table 4**).

**Table 4:** Choice by consumption and risk confound model.

Predictor variable	B	CI	*P*-value	*P*-adj
Actual value	−.007	[−.008, −.005]	**<.001**	**.002**
Outcome type (bad vs. good)	−.055	[−.092, −.017]	**.006**	**.01**
Outcome type (mid vs. good)	−.036	[−.068, −.001]	**.04**	**.05**
# videos consumed	−.001	[−.002, −.001]	** <.001**	**.002**
Risk	−.002	[−005, .000]	.07	.07

B, unstandardized coefficient; CI, confidence interval; P-adj, FDR-adjusted p-values.

Bolded text indicates p-values that are below 0.05.

#### Is the Effect Simply Due to Seeking Gains and Avoiding Losses?

The analyses above showed that the effect of risky trials on subsequent choices depended on the unexpected costs of the trial: a bad outcome meant spending more time than expected and was therefore a loss (worse than expected), while a good outcome meant spending less time than expected and was therefore a gain (better than expected). To test whether this was a general property of unexpected gains and losses, we turned to variability in the ratings within each gallery. While all the videos within a gallery were similar (e.g., cute videos of kittens), each individual video was different. Thus, subjects had an expectation of video quality based on their gallery preferences, but observed a specific video on completion of the delay that might have been better or worse than the average. This produced variability in the post-video ratings: for example, seeing a video rated worse than average was effectively a loss, while a video rated better than average was effectively a gain. In general, we can consider video ratings themselves as a measure of gain/loss.

**Figure 6:**
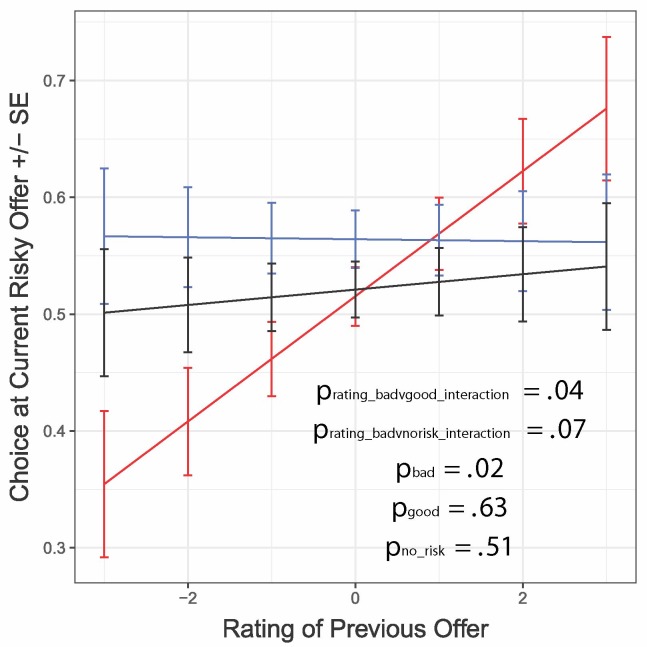
Interaction between previous outcome type and rating when predicting choices on subsequent risky offers. Following receipt of the bad outcome, subjects were more risk-averse after lower-rated videos and more risk seeking after higher-rated videos; no association was detected for the other conditions; ratings are mean-centered. Error bars represent within-subject standard errors.

We used a *secondary model* to test whether video ratings directly guided future choices under the different conditions of interest (**Figure 6**; **Table 5**). We found trend-level interactions between outcome and rating (*p*-adj = 0.06, *p*-adj = 0.09): following risky losses, relatively lower ratings predicted risk aversion, whereas relatively higher ratings yielded risk-seeking behaviors (β = 0.040, CI = [0.005, 0.071], *p* = 0.02). We did not detect associations between video ratings and subsequent choice following good outcomes (β = 0.009, CI = [-0.028, 0.039], *p* = 0.63) or non-risky trials (β = 0.011, CI = [-0.017, 0.043], *p* = 0.51). Thus, ratings only produced changes in risk seeking if in the context of bad outcomes on risky trials (akin to a win–stay/lose–shift strategy) (44). This implies that there was something different about risky losses that went beyond the mere experience of a less enjoyable reward (since a good outcome of the risky trial leading to a poorly rated video was still a loss but did not impact subsequent reward pursuit). We note that these effects remained when accounting for the number of prior videos consumed.

**Table 5:** Choice by rating integrated model.

Predictor variable	B	CI	*P*-value	*P*-adj
Actual value	−.006	[−.008, −.004]	**<.001**	**.004**
Rating	.040	[.005, .073]	**.03**	.06
Outcome type (good vs. bad)	.049	[.015, .090]	**.01**	**.03**
Outcome type (non-risk vs. bad)	.006	[−.028, .039]	.74	.74
Actual value × rating	.002	[.000, .004]	.12	**.14**
Rating × outcome type (good vs. bad)	−.055	[−.110, −.004]	.04	.06
Rating × outcome type (non-risk vs. bad)	−.047	[−.102, .001]	.07	.09

B, unstandardized coefficient; CI, confidence interval; P-adj, FDR-adjusted p-values.

Bolded text indicates p-values that are below 0.05.

### Failure to Learn from Loss After Risk Correlated With Externalizing Traits

**Figure 7:**
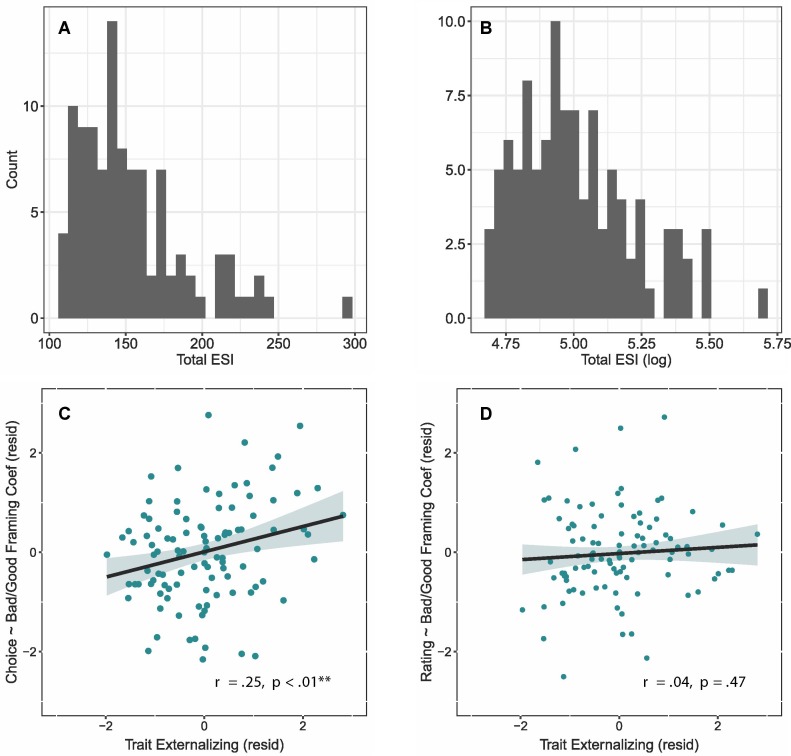
Externalizing Spectrum Inventory distributions and risky loss associations. Distribution of scores from the Externalizing Spectrum Inventory (ESI), shown as raw values **(A)** and logged values **(B)**. **(C)** Relationship between trait-level externalizing and the likelihood of accepting a risk offer after previously receiving the bad outcome. Impulsive subjects showed less risk aversion in response to bad outcomes. **(D)** Relations between trait-level externalizing and immediate likability ratings (mean-centered). Impulsive subjects did not differ in their ratings following bad outcomes. **p < 0.01.

To explore the importance of personality traits to risky decision-making, we investigated whether individuals scoring high on the Externalizing Spectrum Inventory (ESI, 19, which measures a range of impulsive, substance use, and aggressive behaviors) were less influenced by risk when making choices. **Figure 7A and B** shows the distribution of observed ESI values. Many subjects who typically score high on externalizing inventories, such as chronic smokers and individuals at risk for addiction, have been seen to be less influenced by risk when making choices (45, 46). We examined whether such individuals exhibited similar risk-induced effects on reward valuation (i.e., video ratings).

Informed by the group-level model, we computed a parameter that compared a subject’s likelihood of accepting a risky offer after receipt of a good versus bad outcome on the prior trial. Individuals scoring high on the ESI showed an inverse pattern to that observed at the group level (partial *r* = 0.25, *p* = 0.008; **Figure 7C**); these individuals were more likely to accept a risky offer after having just received a bad outcome, signifying a potential deficiency in learning from risky losses. In contrast, the association between outcome type and ratings was unrelated to ESI scores (partial *r* = 0.04, *p* = 0.47; **Figure 7D**).^4^ Together, these results indicate that these externalizing traits affected individual differences in reward pursuit but not reward valuation.

Based on the group-level results above, we used *follow-up partial correlations* to probe whether reward pursuit was related to the broader *substance abuse* subfactor (versus *general disinhibition* and *callous aggression)*, as well as its underlying problem subscales (i.e., *alcohol problems*, *marijuana problems*, *drug problems*. We computed one-tailed robust correlations (i.e., assuming more risk seeking after bad outcomes) and report original and FDR-adjusted p-values that account for the six follow-up correlations.

**Figure 8:**
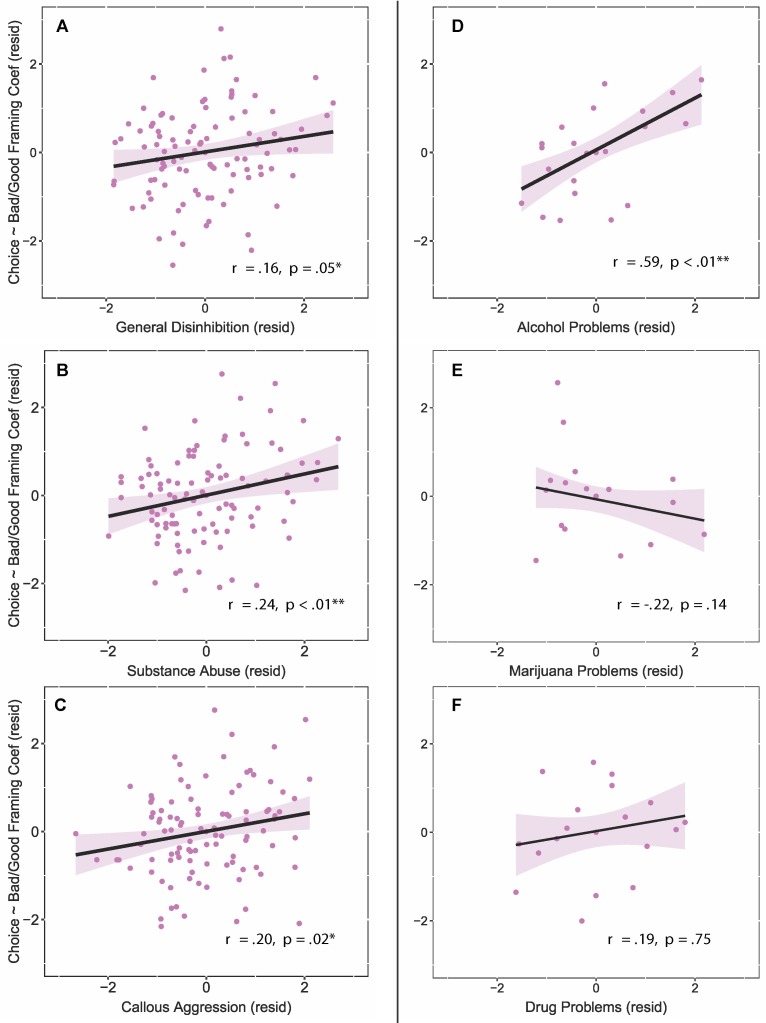
Risky losses influence each ESI subfactor and alcohol problems. The left trio of panels **(A, B, C)** shows the correlation between each ESI subfactor and the likelihood of accepting a risk offer after previously receiving a bad outcome. More externalizing subjects showed less risk aversion in response to bad outcomes for each subfactor. The right trio of panels **(D, E, F)** shows correlations with the three problem subscales of the substance abuse subfactor; more problematic alcohol use was associated with less risk aversion after a bad outcome. *p < 0.05; **p < 0.01.

Our results revealed that two of the three ESI subcomponents were correlated with reward pursuit when accounting for multiple comparisons (general disinhibition partial *r* = 0.16, *p* = 0.05, *p*-adj = 0.08; substance abuse partial *r* = 0.24, *p* = 0.005, *p*-adj = 0.01; callous aggression partial *r* = 0.20, *p* = 0.02, *p*-adj = 0.05; **Figure 8A–C**); however, only substance abuse remained a significant predictor when accounting for the other two subcomponents (partial *r* = 0.18, *p* = 0.03). Further, for individuals endorsing alcohol problems, we found a positive association between reward pursuit after risk and the alcohol problem subscale (*r* = 0.59, *p* = 0.004, *p*-adj = 0.01; **Figure 8D**), versus no association with the marijuana (*r* = −.22, *p* = 0.14, *p*-adj = 0.17; **Figure 8E**) and drug problem (*r* = 0.19, *p* = 0.75, *p*-adj = 0.75; **Figure 8F**) subscales.

### Discounting Rates Did Not Explain the Effects of Externalizing Traits on Reward Pursuit

We computed a series of follow-up robust partial correlations to compare Web-Surf Task–derived metrics with those from a traditional discounting task. The first two correlations predicted total ESI scores from the log-transformed delay and probability k-values, while controlling for age, sex, and ethnicity. Here we found that discounting rates did not significantly predict externalizing (delay k-value: partial *r* = 0.08, *p* = 0.46; probability k-value: partial *r* = 0.02, *p* = 0.88). We then checked whether k-values were related specifically to the substance abuse subfactor, given null associations with the total score and our interest in addiction liability. Similarly, k-values were unrelated to substance abuse (delay k-value: partial *r* = 0.05, *p* = 0.68; probability k-value: partial *r* = −.07, *p* = 0.57). Lastly, we tested whether the subject-level coefficient from the Web-Surf Task that indicated sequencing responses following receipt of a good versus bad outcome still predicted ESI scores, after controlling for the two k-values and additional covariates. Importantly, the Web-Surf Task parameter capturing reward pursuit following risk still predicted ESI scores, even when accounting for the two k-values (partial *r* = 0.25, *p* = 0.03).

## Discussion

We assessed the effects of wins and losses on reward valuation and reward pursuit in a new risk variant of the Web-Surf Task. We found that receipt of the bad outcome on a risky gamble influenced both reward valuation and reward pursuit, but in opposite directions; that is, bad outcomes after risk led to reduced reward pursuit and higher reward valuation. Follow-up analyses showed that offer value impacted these effects, whereby low-value risky losses led to risk aversion and lower-than-normal reward valuations, while high-value losses led to risk seeking and higher-than-normal reward valuations. Subjects were also slower to make decisions after bad outcomes, which points to a post-error slowing effect. There was no impact on willingness to take risks following wins after risk situations (better than expected) or after non-risky control trials. Thus, there was something unique about situations in which subjects lost after deciding to take a risk that increased plasticity in risk-seeking behaviors. Importantly, we also found that trait-level externalizing, particularly substance use tendencies, tracked whether these situations influenced future decisions. Externalizing behaviors were not better explained by performance on a traditional discounting task, highlighting the value of foraging behaviors in capturing substance use disorder vulnerabilities.

In line with our hypotheses and prospect theory (13), the *framing* of an offer relative to the mid-point (versus its absolute value) impacted subsequent reward pursuit and reward valuation. That is, whether an outcome was good or bad relative to the mid-point influenced a subject’s performance regardless of whether he or she took the correct action, as determined by comparing risky and non-risky offers of equivalent value, where non-risky outcomes did not influence performance. Framing effects were also not better explained by global trends in risk aversion. Global trend analyses showed that subjects accepted fewer risky deals as the session progressed, which could suggest that subjects become more sensitive to punishment over time and/or that they experienced reward satiety from ongoing reward exposure. Regardless, the tendency to turn down risky deals following bad outcomes remained when accounting for global risk-aversion trends, highlighting the impact of framing effects on risky choices above and beyond other influences.

Choosing to accept a risky deal and finding oneself in the bad outcome, i.e., with a longer delay than expected, may also be seen as a regret-inducing situation. Constructs of regret suggest that regret occurs at the intersection of agency and mistake (47, 48), where a subject recognizes that an alternate choice (counterfactual) would have led to a better outcome (49). Counterfactually, the subject could have “just skipped it” if only they had known they were going to get the bad deal. A similar phenomenon has been found in mice running the Restaurant Row task, in which mice show regret-related behaviors after accepting a deal and then quitting out of it, but not after spending the same amount of time deliberating over the offer before skipping it (50).

The finding for slowed reaction times after risky losses is consistent with observations in humans of post-error slowing (51–53) but contrasts with findings that rats and mice respond more quickly to the next trial after making a mistake of their own agency (8, 50). There remain several differences between these tasks: 1) the human task presented here included chance and risk, while the rodent tasks were deterministic; 2) humans had brief pre-training, while rodents had months of training; and 3) humans were working for luxury items (videos), while rodents were working for their basic necessities (food intake for the day). And because rodents had a fixed amount of time to consume their meal, there was potentially more impetus to move quickly and consume more food before time ran out. Of course, it is also possible that there could be a species difference in how humans and rodents respond to these tasks, e.g., cross-species divergences in self-evaluation processes following loss could contribute to the observed reaction time differences, although given the similarities recently seen in their response to deliberation and sunk costs (9), this may be less likely. Whether this post-error response inconsistency arises from cross-species differences in response to regret or unique task attributes remains unknown and will have to be left for future study. One possibility is that “regret” is more complicated and that there are differences between realizing that you made a mistake in a situation in which you had all the necessary information to make a better decision versus taking a risk only to find that the answer is not what you hoped for.

Our analyses also revealed that risky losses had an opposite impact on reward valuation, whereby subjects liked videos that followed a bad (long-delay) outcome more than those following a good (short-delay) outcome on risky trials, though we note that the effects of reward valuation were less robust than those for reward pursuit and should be interpreted with caution. These reward valuation results are consistent with economic observations that humans rate outcomes higher when they have spent more on them (54). This suggests that subjects have a backwards-looking view when rating videos that is consistent with explanations of sunk-cost effects seen in human and non-human subjects (9, 55, 56) and with economic explanations for the effect of anticipation on subsequent reward valuation (57). A desire to reduce cognitive dissonance, an aversive mental state that occurs when there is a discrepancy between behavior and attitude (58), could also explain higher ratings following bad outcomes. That is, subjects may have been trying to alter their attitude as a means to reduce psychological discomfort (59).

A key result from this study is that individuals exhibiting greater externalizing disorder vulnerability were more likely to accept a risky offer after receipt of a bad outcome. Critically, our findings were strongest for the substance abuse subfactor, and largely, the alcohol problem subscale, which could reflect the nature of an undergraduate sample. This risky decision–externalizing association is consistent with notions that addiction involves continued reward pursuit despite negative outcomes (60), and could reflect an inability to learn from mistakes (61). These results also speak to dimensional models of psychopathology, given that behavior is correlated with externalizing problems even in the absence of clinical diagnoses.

Compared to reward pursuit, we saw no relation between externalizing and reward evaluation following regret, suggesting that externalizing may have different associations with different facets of the decision process. One hypothesis is that high externalizers do not show differentiation in reward valuation because of a tendency to respond in a socially conforming manner. For instance, prior research suggests that striatal dopamine availability is a common link between the tendency to “fake good,” i.e., respond in a socially desirable way (62, 63), and impulsivity (64). It is then possible that high-externalizing subjects may conform to the socially expected pattern when evaluating rewards. Similarly, externalizing problem behavior is highly related to cognitive distortions, which is an umbrella term that includes the rationalization (or neutralization) of deviant behavior (65). Here, high-externalizing subjects may rationalize their bad decisions with positive ratings. Future research could directly test these theories by including scales that measure socially desirable responding (e.g., the Marlowe–Crowne Social Desirability Scale; 66) or pre-conscious rationalization (65).

Our data could be explained in part by differences in temporal attention, whereby reward valuation is done by looking backwards, while changes in reward pursuit are done by looking forwards. This leads to a key question of whether these two processes are linked. We found them linked in typical subjects, but our individual-differences analyses revealed that these effects occur through separable processes: more externalizing individuals showed comparable effects of risk on reward valuation but did not subsequently modulate their reward pursuit following regret. In fact, **Figure 4** suggests that people scoring high on the ESI may even show the opposite effect, becoming risk seeking after regret-inducing instances. These results are consistent with application of the temporal attention hypothesis to delay discounting, in which a preference for immediate rewards among individuals with addiction is due to a narrowing of temporal attention (67); perhaps high-externalizing subjects have a narrowed attention window that leaves valuation of recent consummatory experiences intact but reduces their capacity to evaluate distal outcomes.

As noted above, externalizing tendencies were not associated with performance on a traditional discounting task. This result diverges from established links between substance abuse and discounting (68, 69). One possible explanation is that steeper discounting is more strongly tied to *current* substance abuse versus a *liability* towards substance abuse. For instance, while steeper discounting rates are observed in chronic nicotine users, discounting rates have been shown to normalize among ex-smokers (70, 71). Gowin et al. (69) observed similar results, where individuals with current alcohol use disorder (AUD) had steeper discounting rates than healthy controls, but individuals with past AUD showed no difference from controls. The fact that our sample includes both individuals with a substance use/abuse history and individuals who are prone to substance use may have reduced our likelihood of capturing such a link. This explanation is in line with Isen et al. (72), who found that hypothetical delay-discounting behaviors did not predict latent trait-externalizing tendencies as similarly measured with the ESI. This again suggests that there may be weaker relationships between discounting behaviors and externalizing liability.

### Limitations

A recognized limitation of the current study is the use of an undergraduate sample that was not specifically recruited based on substance use history. However, the fact that we still detected foraging–substance use relations suggests that the task is sensitive to behaviors that are likely present even at the lower end of the externalizing spectrum; this study also provides a set of foundational findings that can be tested in a confirmatory manner to clarify whether reward pursuit during foraging similarly tracks recreational and problematic substance use in the broader community and among individuals with varying levels of usage. Another limitation is the lack of consumption or craving measurements, as these factors could moderate the observed effects. We also acknowledge that the consequences of a risky loss on the Web-Surf Task is small relative to real-life consequences like filing for bankruptcy, losing transportation options following a DUI, or being imprisoned; but if we find substance use associations when the stakes are low, we might expect greater effects as substance use becomes more chronic and/or problematic.

### Conclusions

Our results suggest a dissociation among individuals with greater substance use disorder vulnerability: costly experiences serve to enhance reward value but did not impact subsequent reward pursuit following regret. Taken together, a blunted sense of regret may result in an overvaluation of risky losses that in turn drives the continued pursuit of risky endeavors. Future work will assess the impact of risky losses while foraging in clinical samples.

## Ethics Statement

The University of Minnesota Institutional Review Board approved the study procedures, and all subjects provided written informed consent.

## Author Contributions

SA, AR, and AM designed the experiment. AR and AM supervised the project. SA carried out the experiments and analyzed the data. SA, AR, and AM wrote the manuscript.

## Funding

This work was supported by grants from the National Institute on Drug Abuse (NIDA) to SA (F31-DA040335-02) and AR (R01-DA030672). The writing of this manuscript was supported by the Department of Veterans Affairs Office of Academic Affiliations; the Advanced Fellowship Program in Mental Illness Research and Treatment; and the Department of Veterans Affairs Sierra Pacific Mental Illness Research, Education, and Clinical Center (MIRECC).

## Conflict of Interest Statement

The authors declare that the research was conducted in the absence of any commercial or financial relationships that could be construed as a potential conflict of interest.

## Footnotes

1 Missing self-report data for 1 subject.

2 Missing delay- and probability-discounting data for three subjects.

3 We excluded nine subjects with invalid k-values (discounting rates of 0), one subject with a k-value more than 4 standard deviations above the mean, and one subject with nonsystematic data.

4 We excluded one subject with a coefficient less than 4 standard deviations below the mean.

## References

[1] Tom SM Fox CR Trepel C Poldrack RA The neural basis of loss aversion in decision-making under risk. Science (2007) 315:515–8. 10.1126/science.1134239 17255512

[2] Connolly T Zeelenberg M Regret in decision making. Curr Dir Psychol Sci (2002) 11:212–6. 10.1111/1467-8721.00203

[3] Coricelli G Dolan RJ Sirigu A Brain, emotion and decision making: the paradigmatic example of regret. Trends Cogn Sci (2007) 11(6);258–65. 10.1016/j.tics.2007.04.003 17475537

[4] Loomes G Sugden R Regret theory: an alternative theory of rational choice under uncertainty. Econ J (1982) 92:805. 10.2307/2232669

[5] Chiu PH Lohrenz TM Montague PR Smokers’ brains compute, but ignore, a fictive error signal in a sequential investment task. Nat Neurosci (2008) 11:514–20. 10.1038/nn2067 18311134

[6] Sweis BM Redish AD Thomas MJ Prolonged abstinence from cocaine or morphine disrupts separable valuations during decision conflict. Nat Commun (2018) 1–9. 10.1038/s41467-018-04967-2 29955073 PMC6023899

[7] Abram SV Breton Y-A Schmidt B Redish AD MacDonald AW The Web-Surf Task: a translational model of human decision-making. Cogn Affect Behav Neurosci (2016) 16(1):37–50. 10.3758/s13415-015-0379-y 26377334 PMC4761272

[8] Steiner AP Redish AD Behavioral and neurophysiological correlates of regret in rat decision-making on a neuroeconomic task. Nat Neurosci (2014) 17(7):995–1002. 10.1038/nn.3740 24908102 PMC4113023

[9] Sweis BM Abram SV Schmidt BJ Seeland KD MacDonald AW Thomas MJ Sensitivity to “sunk costs” in mice, rats, and humans. Science (2018) 361:178–81. 10.1126/science.aar8644 PMC6377599 30002252

[10] Stephens DW Decision ecology: foraging and the ecology of animal decision making. Cogn Affect Behav Neurosci (2008) 8(4):475–84. 10.3758/CABN.8.4.475 19033242

[11] Feighery EC Schleicher NC Boley Cruz T Unger JB An examination of trends in amount and type of cigarette advertising and sales promotions in California stores, 2002–2005. Tob Control (2008) 17:93–8. 10.1136/tc.2007.022046 18303088

[12] De Wit H Impulsivity as a determinant and consequence of drug use: a review of underlying processes. Addict Biol (2009) 14(1):22–31. 10.1111/j.1369-1600.2008.00129.x 18855805 PMC3640851

[13] Kahneman D Tversky A Prospect theory: an analysis of decision under risk. Econometrica (1979) 47:263–92. 10.1111/j.1536-7150.2011.00774.x

[14] Phillips JG Currie J Ogeil RP Consumption and foraging behaviors for common stimulants (nicotine, caffeine). J Addict Dis (2016) 35:15–21. 10.1080/10550887.2015.1094721 26555360

[15] Kendler KS Prescott C Myers J Neale MC The structure of genetic and environmental risk factors for common psychiatric and substance use disorders in men and women. Arch Gen Psychiatry (2003) 60(9):929–37. 10.1001/archpsyc.60.9.929 12963675

[16] Krueger RF Hicks BM Patrick CJ Carlson SR Iacono WG McGue M Etiologic connections among substance dependence, antisocial behavior and personality: modeling the externalizing spectrum. J Abnorm Psychol (2002) 111(3):411–24. 10.1037//0021-843X.111.3.411 12150417

[17] Krueger RF Markon KE Patrick CJ Iacono WG Externalizing psychopathology in adulthood: a dimensional-spectrum conceptualization and its implications for DSM-V. J Abnorm Psychol (2005) 114:537–50. 10.1037/0021-843X.114.4.537 PMC2242352 16351376

[18] Lauriola M Panno A Levin IP Lejuez CW Individual differences in risky decision making: a meta-analysis of sensation seeking and impulsivity with the Balloon Analogue Risk Task. J Behav Decis Mak (2014) 27(1):20–36. 10.1002/bdm.1784

[19] Krueger RF Markon KE Patrick CJ Benning SD Kramer MD Linking antisocial behavior, substance use, and personality: an integrative quantitative model of the adult externalizing spectrum. J Abnorm Psychol (2007) 116(4):645–66. 10.1037/0021-843X.116.4.645 PMC2242625 18020714

[20] Blonigen DM Patrick CJ Gasperi M Steffen B Ones DS Arvey RD Delineating the construct network of the personnel reaction blank: associations with externalizing tendencies and normal personality. Psychol Assess (2011) 23:18–30. 10.1037/a0021048 21171783 PMC3058622

[21] Brislin SJ Yancey JR Perkins ER Palumbo IM Drislane LE Salekin RT Callousness and affective face processing in adults: behavioral and brain-potential indicators. Personal Disord (2018) 9:122–32. 10.1037/per0000235 PMC5511780 28095001

[22] Hall JR Bernat EM Patrick CJ Externalizing psychopathology and the error-related negativity. Psychol Sci (2007) 18:326–33. 10.1111/j.1467-9280.2007.?01899.x PMC2242425 17470258

[23] Meehan KB De Panfilis C Cain NM Clarkin JF Effortful control and externalizing problems in young adults. Pers Individ Dif (2013) 55:553–8. 10.1016/j.paid.2013.04.019

[24] Patrick CJ Kramer MD Krueger RF Markon KE Optimizing efficiency of psychopathology assessment through quantitative modeling: development of a brief form of the Externalizing Spectrum Inventory. Psychol Assess (2013) 25(4):1332–48. 10.1037/a0034864 PMC4100945 24320765

[25] Zucker RA Anticipating problem alcohol use developmentally from childhood into middle adulthood: what have we learned? Addiction (2008) 103:100–8. 10.1111/j.1360-0443.2008.02179.x PMC2593849 18426543

[26] Benjamini Y Hochberg Y Controlling the false discovery rate: a practical and powerful approach to multiple testing. J Royal Stat Soc B (1995) 57(1):289–300. 10.1111/j.2517-6161.1995.tb02031.x

[27] Hadfield JD MCMC methods for multi-response generalized linear models. J Stat Softw (2010) 33:1–22. 10.1002/ana.22635 20808728 PMC2929880

[28] Bates D Maechler M Walker S Package “lme4”: linear mixed-effects models using “Eigen” and S4. CRAN Repository (2016) 1–113. 10.18637/jss.v067.i01

[29] Lenth RV Least-squares means: the R package lsmeans. J Stat Softw (2016) 69(1):1–33. 10.18637/jss.v069.i01

[30] Hahn PR (n.d). Statistical formula notation in R. Retrieved from https://faculty.chicagobooth.edu/richard.hahn/teaching/formulanotation.pdf.

[31] de Wit H Flory JD Acheson A McCloskey M Manuck SB IQ and nonplanning impulsivity are independently associated with delay discounting in middle-aged adults. Pers Individ Dif (2007) 42(1):111–21. 10.1016/j.paid.2006.06.026

[32] Eaton NR Keyes KM Krueger RF Balsis S Skodol AE Markon KE An invariant dimensional liability model of gender differences in mental disorder prevalence: evidence from a national sample. J Abnorm Psychol (2012) 121(1):282–8. 10.1037/a0024780 PMC3402021 21842958

[33] Kramer MD Krueger RF Hicks BM The role of internalizing and externalizing liability factors in accounting for gender differences in the prevalence of common psychopathological syndromes. Psychol Med (2008) 38:51–61. 10.1017/S0033291707001572 17892625

[34] Romer D Hennessy M A biosocial-affect model of adolescent sensation seeking: the role of affect evaluation and peer-group influence in adolescent drug use. Prev Sci (2007) 8:89–101. 10.1007/s11121-007-0064-7 17286212

[35] Chen P Jacobson KC Developmental trajectories of substance use from early adolescence to young adulthood: gender and racial/ethnic differences. J Adolesc Health (2012) 50:154–63. 10.1016/j.jadohealth.?2011.05.013 PMC3264901 22265111

[36] Anokhin AP Golosheykin S Grant JD Heath AC Heritability of delay discounting in adolescence: a longitudinal twin study. Behav Genet (2011) 41(2):175–83. 10.1007/s10519-010-9384-7 PMC3036802 20700643

[37] Olson EA Hooper CJ Collins P Luciana M Adolescents’ performance on delay and probability discounting tasks: contributions of age, intelligence, executive functioning, and self-reported externalizing behavior. Pers Individ Dif (2007) 43(7):1886–97. 10.1016/j.paid.2007.06.016 PMC2083651 18978926

[38] Reynolds B A review of delay-discounting research with humans: relations to drug use and gambling. Behav Pharmacol (2006) 17(8):651–67. 10.1097/FBP.0b013e3280115f99 17110792

[39] Ainslie G Haslam N Hyperbolic discounting. In Loewenstein G Elster J (Eds.), Choice over Time. New York: Russell Sage Foundation p. 57–92.

[40] Bickel WK Jarmolowicz DP Mueller ET Koffarnus MN Gatchalian KM Excessive discounting of delayed reinforcers as a trans-disease process contributing to addiction and other disease-related vulnerabilities: emerging evidence. Pharmacol Ther (2012) 134(3):287–97. 10.1016/j.pharmthera.2012.02.004 PMC3329584 22387232

[41] Johnson MW Bickel WK An algorithm for identifying nonsystematic delay-discounting data. Exp Clin Psychopharmacol (2008) 16:264–74. 10.1037/1064-1297.16.3.264 PMC2765051 18540786

[42] Plassmann H O’Doherty J Shiv B Rangel A Marketing actions can modulate neural representations of experienced pleasantness. Proc Natl Acad Sci U S A (2008) 105(3):1050–54. 10.1073/pnas.0706929105 PMC2242704 18195362

[43] Sweis BM Abram SV Schmidt BJ Seeland KD MacDonald AW Thomas MJ Redish AD Sensitivity to “sunk costs” in mice, rats, and humans. Science (2018b) 361:178–81. 10.1126/science.aar8644 PMC6377599 30002252

[44] Worthy DA Hawthorne MJ Otto AR Heterogeneity of strategy use in the Iowa gambling task: a comparison of win–stay/lose–shift and reinforcement learning models. Psychon Bull Rev (2013) 20(2):364–71. 10.3758/s13423-012-0324-9 23065763

[45] Benegal V Antony G Venkatasubramanian G Jayakumar PN Gray matter volume abnormalities and externalizing symptoms in subjects at high risk for alcohol dependence. Addict Biol (2007) 12:122–32. 10.1111/j.1369-1600.2006.00043.x 17407506

[46] Carroll AJ Sutherland MT Salmeron BJ Ross TJ Stein EA Greater externalizing personality traits predict less error-related insula and anterior cingulate cortex activity in acutely abstinent cigarette smokers. Addict Biol (2015) 20:377–89. 10.1111/adb.12118 PMC4057361 24354662

[47] Van Dijk WW Zeelenberg M Investigating the appraisal patterns of regret and disappointment. Motiv Emot (2002) 26:321–31. 10.1023/A:1022823221146

[48] Zeelenberg M Van Dijk WW Manstead ASR Van Der Pligt J The experience of regret and disappointment. Cogn Emot (1998) 12:221–30. 10.1080/026999398379727 9719660

[49] Bell DE Regret in decision making under uncertainty. Oper Res (1982) 30:961–81. 10.1287/opre.30.5.961

[50] Sweis BM Thomas MJ Redish AD Mice learn to avoid regret. PLoS Biol (2018) 16(6) 1–21. 10.1371/journal.pbio.2005853 PMC6013153 29927938

[51] Dutilh G Vandekerckhove J Forstmann BU Keuleers E Brysbaert M Wagenmakers EJ Testing theories of post-error slowing. Atten Percept Psychophys (2012) 74(2):454–65. 10.3758/s13414-011-0243-2 PMC3283767 22105857

[52] Laming D Choice reaction performance following an error. Acta Psychol (1979) 43(3):199–224. 10.1016/0001-6918(79)90026-X 495175

[53] Rabbitt P Rodgers B What does a man do after he makes an error? an analysis of response programming. Q J Exp Psychol (1977) 29(4):727–43. 10.1080/14640747708400645

[54] Cunha M Caldieraro F Sunk-cost effects on purely behavioral investments. Cogn Sci (2009) 33:105–13. 10.1111/j.1551-6709.2008.01005.x 21585465

[55] Aronson E The effect of effort on the attractiveness of rewarded and unrewarded stimuli. J Abnorm Psychol (1961) 63:375–80. 10.1037/h0046890 13862515

[56] Wikenheiser AM Stephens DW Redish AD Subjective costs drive overly patient foraging strategies in rats on an intertemporal foraging task. Proc Natl Acad Sci (2013) 110(20):8308–13. 10.1073/pnas.1220738110 PMC3657802 23630289

[57] Loewenstein G Anticipation and the valuation of delayed consumption. Econ J (1987) 97(387):666. 10.2307/2232929

[58] Festinger L An introduction to the theory of dissonance. a theory of cognitive dissonance. Stanford, CA: Stanford University Press (1957). 10.1037/10318-001

[59] Elliot AJ Devine PG On the motivational nature of cognitive dissonance: dissonance as psychological discomfort. J Pers Soc Psychol (1994) 67(3):382–94. 10.1037/0022-3514.67.3.382

[60] Hyman SE Addiction: a disease of learning and memory. Am J Psychiatry (2005) 162(8):1414–22. 10.1176/appi.ajp.162.8.1414 16055762

[61] Bechara A Risky business: emotion, decision-making, and addiction. J Gambl Stud (2003) 19:23–51. 10.1023/A:1021223113233 12635539

[62] Egerton A Rees E Bose SK Lappin JM Stokes PRA Turkheimer FE Truth, lies or self-deception? Striatal D2/3 receptor availability predicts individual differences in social conformity. NeuroImage (2010) 53:777–81. 10.1016/j.neuroimage.2010.06.031 20558302

[63] Reeves SJ Mehta MA Montgomery AJ Amiras D Egerton A Howard RJ Striatal dopamine (D2) receptor availability predicts socially desirable responding. NeuroImage (2007) 34:1782–9. 10.1016/j.neuroimage.2006.10.042 17188897

[64] Anderson BA Kuwabara H Wong DF Courtney SM Density of available striatal dopamine receptors predicts trait impulsiveness during performance of an attention-demanding task. J Neurophysiol (2017) 117(1):64–8. 10.1152/jn.00125.2017 PMC5494363 28356473

[65] Helmond P Overbeek G Brugman D Gibbs JC A meta-analysis on cognitive distortions and externalizing problem behavior: associations, moderators, and treatment effectiveness. Crim Justice Behav (2015) 42(3):245–62. 10.1177/0093854814552842

[66] Crowne DP Marlowe D A new scale of social desirability independent of psychopathology. J Consult Psychol (1960) 24:349–54. 10.1037/h0047358 13813058

[67] McClure SM Bickel WK A dual-systems perspective on addiction: contributions from neuroimaging and cognitive training. Ann N Y Acad Sci (2014) 1327:62–78. 10.1111/nyas.12561 25336389 PMC4285342

[68] Bickel WK Marsch LA Toward a behavioral economic understanding of drug dependence: delay discounting processes. Addiction (2001) 96:73–86. 10.1046/j.1360-0443.2001.961736.x 11177521

[69] Gowin J Sloan ME Swan JE Momenan R Ramchandani VA The relationship between delay discounting and alcohol dependence in individuals with and without comorbid psychopathology. Psychopharmacology (2019) 236(2):775–85. 10.1007/s00213-018-5113-3 PMC6401281 30456539

[70] Bickel WK Odum AL Madden GJ Impulsivity and cigarette smoking: delay discounting in current, never, and ex-smokers. Psychopharmacology (1999) 146:447–54. 10.1007/PL00005490 10550495

[71] Secades-Villa R Weidberg S García-Rodríguez O Fernández-Hermida JR Yoon JH Decreased delay discounting in former cigarette smokers at one year after treatment. Addict Behav (2014) 39(6):1087–93. 10.1016/j.addbeh.2014.03.015 24661901

[72] Isen JD Sparks JC Iacono WG Predictive validity of delay discounting behavior in adolescence: a longitudinal twin study. Exp Clin Psychopharmacol (2014) 22(5):434–43 10.1037/a0037340 PMC4180746 24999868

[73] Abram SV Towards a translational model of decision-making: findings from the Web-Surf Task. University of Minnesota (2017).

